# Transcriptional data analysis reveals the association between infantile hemangiomas and venous malformations

**DOI:** 10.3389/fgene.2022.1045244

**Published:** 2022-10-19

**Authors:** Biao Huang, Ping Zhang, Yuan-Yuan Zhong, Kuan Wang, Xiao-Ming Chen, Dao-Jiang Yu

**Affiliations:** ^1^ Department of Plastic and Burn Surgery, The Second Affiliated Hospital of Chengdu Medical College, China National Nuclear Corporation 416 Hospital, Chengdu, China; ^2^ West China School of Basic Medical Sciences and Forensic Medicine, Sichuan University, Chengdu, China; ^3^ Department of Plastic Surgery, The First Affiliated Hospital of Chengdu Medical College, Chengdu, China; ^4^ Department of Health Management Center, The Second Affiliated Hospital of Soochow University, Soochow, China

**Keywords:** infantile hemangiomas, venous malformation, bioinformatics analysis, differentially expressed genes, microRNA

## Abstract

**Background:** Infantile hemangiomas (IH) and venous malformations (VM) are the most common types of vascular abnormalities that seriously affect the health of children. Although there is evidence that these two diseases share some common genetic changes, the underlying mechanisms need to be further studied.

**Methods:** The microarray datasets of IH (GSE127487) and VM (GSE7190) were downloaded from GEO database. Extensive bioinformatics methods were used to investigate the common differentially expressed genes (DEGs) of IH and VM, and to estimate their Gene Ontology (GO) and Kyoto Encyclopedia of Genes and Genomes (KEGG) pathways. Trough the constructing of protein-protein interaction (PPI) network, gene models and hub genes were obtained by using Cytoscape and STRING. Finally, we analyzed the co-expression and the TF-mRNA-microRNA regulatory network of hub genes.

**Results:** A total of 144 common DEGs were identified between IH and VM. Functional analysis indicated their important role in cell growth, regulation of vasculature development and regulation of angiogenesis. Five hub genes (CTNNB1, IL6, CD34, IGF2, MAPK11) and two microRNA (has-miR-141-3p, has-miR-150-5p) were significantly differentially expressed between IH and normal control (*p* < 0.05).

**Conclusion:** In conclusion, our study investigated the common DEGs and molecular mechanism in IH and VM. Identified hub genes and signaling pathways can regulate both diseases simultaneously. This study provides insight into the crosstalk of IH and VM and obtains several biomarkers relevant to the diagnosis and pathophysiology of vascular abnormalities.

## Introduction

Infantile hemangiomas (IH) are the most common benign tumors in early childhood, with an incidence of 4%–10% (Rodríguez Bandera et al., 2021). Although almost every part of the human body can be involved, hemangiomas are seen more frequently in head and neck (Fernández Faith et al., 2021). The hyper-proliferation of IH can cause significant functional and disfiguring consequences such as ulceration, bleeding, and pain. Vascular malformations affect 3% of the population. Venous malformations (VM) are the most common type representing more than 50% of cases (Gallant et al., 2021). VM grows proportionally with the human body and do not spontaneously involute. It is typically characterized by dilated superficial veins, purple vein bubbles, or blue tinge involving the skin (Kunimoto et al., 2022). Medical interventions or surgical treatments are required by 45% of patients with vascular anomalies (Haggstrom et al., 2006). Based on previous experience, the proper treatment of these diseases requires specialists from multiple disciplines, including plastic surgery, pediatrics, dermatology, radiology and vascular surgery.

IH and VM are generally considered to be two distinct diseases, not only in anatomical, histological, and pathophysiological features, but also in clinical presentation. However, they are both vascular anomalies, representing deleterious mutations in vascular development. The commonality among these lesions is their origin in vascular endothelia (Mahajan et al., 2022). TIE2, encoded by TEK, is an endothelial cell-specific receptor tyrosine kinase that is essential for vessel remodeling and the survival of endothelial cells. Limited studies have identified that somatic mutations in exon 17 of the TEK gene were common changes in vascular tumors and vascular malformations (Ye et al., 2011). Due to the association of IH and VM and some common mutations and immune-related factors, some molecular mechanisms might be involved in the development and progression of IH and VM.

Bioinformatics has developed rapidly in recent years, which can be applied to illustrate large and complicated data sets associated with various diseases. Given the reasonable expectations of the development of sequencing technologies, and the decreasing cost barriers, researchers around the world have contributed a large amount of transcriptomic data. Analysis of mRNA transcriptomics may help uncover the biological processes and underlying mechanisms of disease. However, most research teams mainly focus on a single disease, and then delve into the regulatory mechanisms. The characteristics of the shared changes of the vascular anomalies and their relevance remain less explored. Bioinformatics methods enable us to extract multiple related microarrays for further data mining, which contributes to the reuse of information and the discovery of crosstalk between diseases.

**FIGURE 1:**
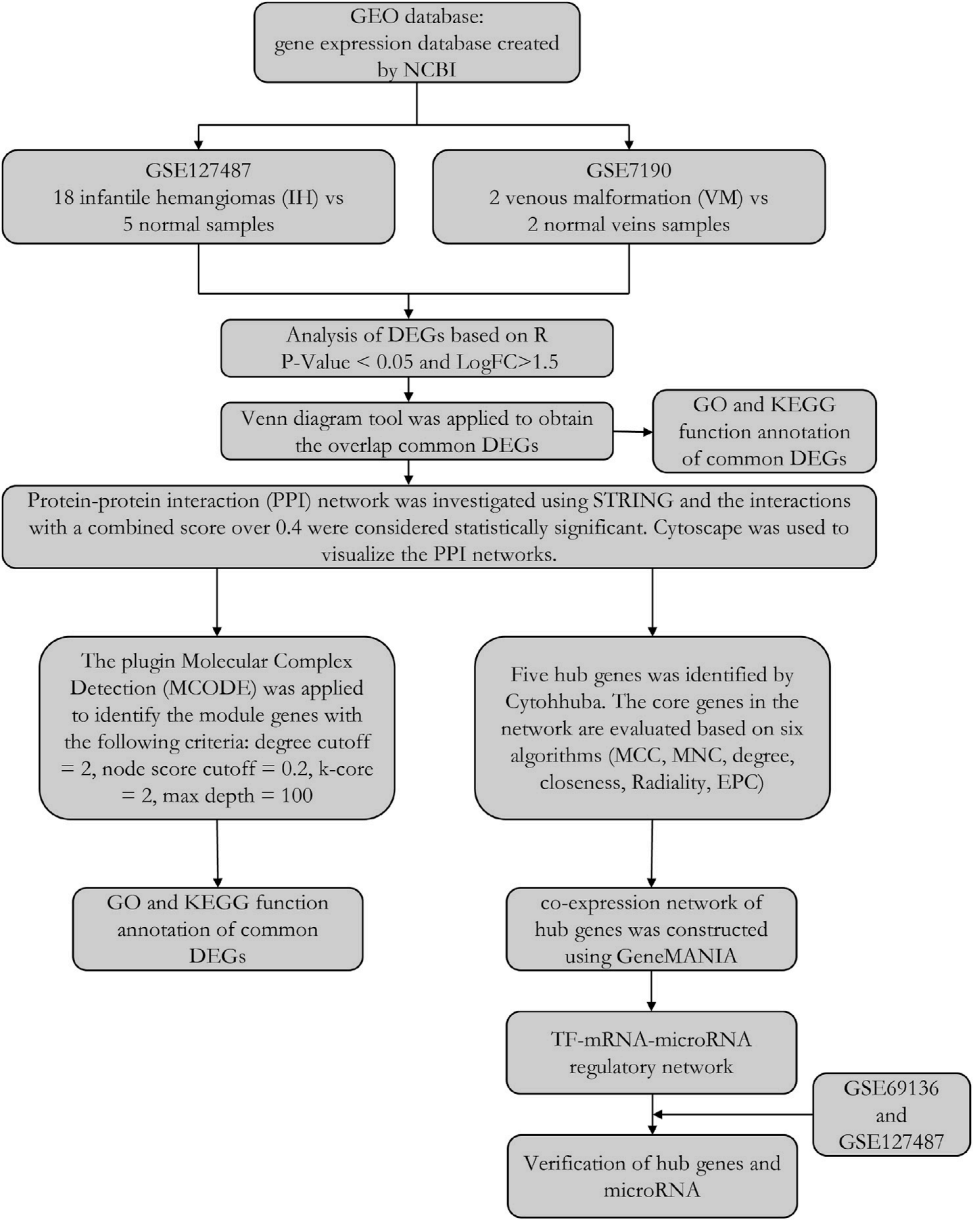
The workflow of this study.

In this study, we tried to identify the common differentially expressed genes (DEGs) between IH and VM patients. Two microarray datasets, GSE7190 and GSE127487, were obtained from GEO database. We analyzed their common DEGs and potential functions. Then, through the construction of PPI network, gene model and hub genes were investigated. Finally, five hub genes were identified and their transcription factor (TF) and microRNA were also traced. The workflow of our study was shown in Figure 1.

## Materials and methods

### Data collection

GEO (http://www.ncbi.nlm.nih.gov/geo) is a gene expression database created by NCBI, which contains high-throughput gene expression data submitted by research institutes worldwide (Barrett et al., 2013). We used the keywords infantile hemangiomas and venous malformation to search for eligible datasets. Among the inclusion criteria were 1) diagnosis of patients with infantile hemangiomas (IH) and venous malformation (VM), 2) detection of gene level in tissue or blood samples. Exclusion criteria were 1) expression data without normal control, and 2) datasets come from *Mus musculus* and Rattus norvegicus. Finally, two microarray datasets were eligible: accession numbers GSE127487 (18 infantile hemangiomas (IH) samples and 5 normal samples, Platforms: GPL10558), GSE7190 (2 venous malformation (VM) samples and 2 normal veins samples, Platforms: GPL1708) (Ebenebe et al., 2007; Gomez-Acevedo et al., 2020). For validation, expression profiles of 12 infantile hemangiomas (IH) patients and 4 normal patients were downloaded from GSE69136 dataset (Platforms: GPL19765) (Strub et al., 2016).

### Analysis of DEGs

Differentially expressed mRNA between infantile hemangiomas (IH) patients and normal skin patients were identified using Limma package of R software. The adj.P.Value <0.05 and LogFC (fold change) > 1.5 or < -1.5 were set as the cutoffs. Used the same procedure to identify differentially expressed genes between venous malformations (VM) and normal veins. Next, the online Venn diagram tool (https://bioinfogp.cnb.csic.es/tools/venny/index.html) was applied to obtain the overlap DEGs among two datasets.

### Function annotation of DEGs

GO (gene ontology) database: the function of genes is divided into three categories: biological process (biological process, BP), cellular components (cellular component, CC), molecular function (molecular function, MF). Using the GO database, we can find out the relationship between the DEGs at the three levels of CC, MF, and BP. Function annotation and Genomes (KEGG) pathways enrichment analysis were performed using DAVID (Huang et al., 2007). P.adjust <0.05 was consider significant.

### Protein-protein interaction network and model construction

Protein-protein interaction network was investigated using the Search Tool for the Retrieval of Interacting Genes/Proteins (STRING: http://string-db.org, version11.5) (Szklarczyk et al., 2015). It can customize PPI networks, as well as functional characterization of user-uploaded gene/measurement sets. The interactions with a combined score over 0.4 were considered statistically significant. Then, we used Cytoscape to visualize the PPI networks (Saito et al., 2012). The plugin Molecular Complex Detection (MCODE) was applied to identify the module genes that interact most closely with the following criteria: degree cutoff = 2, node score cutoff = 0.2, k-core = 2, max depth = 100 (Bandettini et al., 2012). In addition, GO and KEGG analysis of the model genes were performed.

### Identification of hub genes

The plugin Cytohhuba of Cytoscape was applied to identify hub genes in our networks. The top ten core genes in the network are evaluated based on six algorithms (MCC, MNC, degree, closeness, Radiality, EPC). Here, the genes contained in all six algorithms were regarded as hub genes. Subsequently, a co-expression network of hub genes was constructed using GeneMANIA (http://www.genemania.org/), which searches many large, publicly available biological datasets to find related genes (Warde-Farley et al., 2010). These include protein-protein, protein-DNA and genetic interactions, pathways, reactions and phenotypic screening profiles.

### TF-mRNA-microRNA regulatory network

In order to better understand the potential regulatory relationship of these hub genes, we established the TF-mRNA-microRNA regulatory network based on TRRUST (http://www.grnpedia.org/trrust) and Mirwalk (http://mirwalk.umm.uni-heidelberg.de/) database. TRRUST is a manually curated database of human and mouse transcriptional factor regulatory networks. Current version of TRRUST contains 8,444 and 6,552 TF-target regulatory relationships of 800 human TFs (Han et al., 2018). The new version of miRWalk stores predicted data obtained with a maschine learning algorithm including experimentally verified miRNA-target interactions (Sticht et al., 2018).

### Expression verification of hub genes, TFs and microRNA

In order to confirm the credibility of our results, hub genes, TFs and microRNA expression was verified. The microarray dataset GSE34989 and GSE7190 were used for hub gene and TFs expression. Additionally, GSE69136 (12 infantile hemangiomas samples (IH) and 4 normal samples) was used for microRNA verification. Student’s t method was used to test the difference and *p*-value < 0.05 was considered statistically significant.

### Statistical analysis

Two-group comparisons were determined using Student’s *t* test, and multiple group comparisons were conducted using the analysis of variance *t* test. All statistical analyses and pictures (Volcano plots, bubble plots, histograms and ROC) were performed and visualized using R software. *p*-value < 0.05 was considered statistically significant.

## Results

### Identification of common DEGs

**FIGURE 2:**
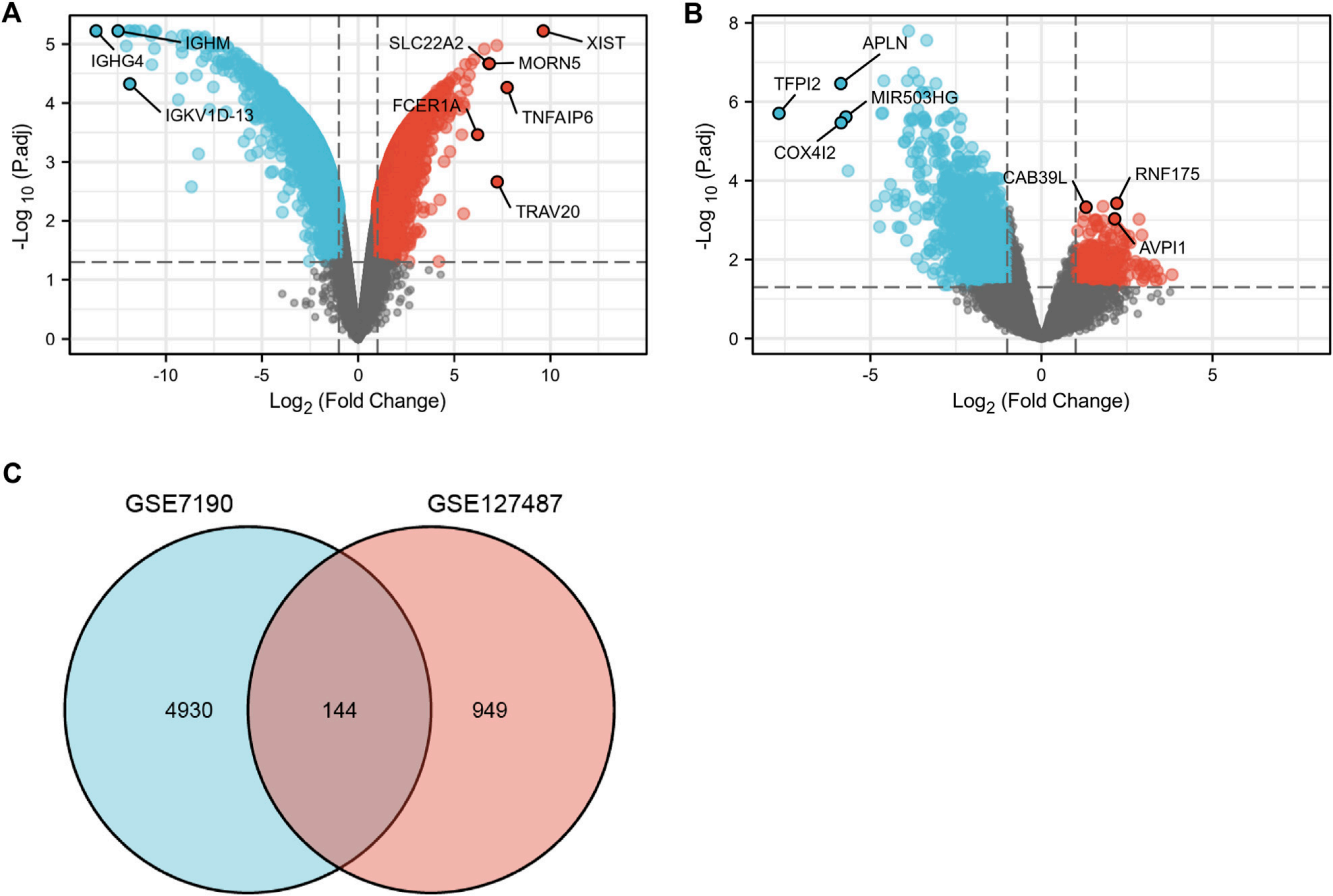
The volcano and Venn diagram. **(A)**, the volcano diagram of GSE7190. **(B)**, The volcano diagram of GSE127487. **(C)**, Venn diagram showed an overlap of 144 common DEGs.

After data standardization and filtering, there were 1,093 DEGs (868 upregulated and 225 downregulated) between infantile hemangiomas group (IH) and normal group (Figure 2A). Additionally, 5,740 DEGs (2,992 upregulated and 2,748 downregulated) were identified between venous malformation group (VM) and normal group (Figure 2B). Through the intersection of the Venn diagram, we obtained 144 overlapped DEGs (107 upregulated and 37 downregulated) in GSE127487 and GSE7190 (Figure 2C).

### Functional analysis of common DEGs

**FIGURE 3:**
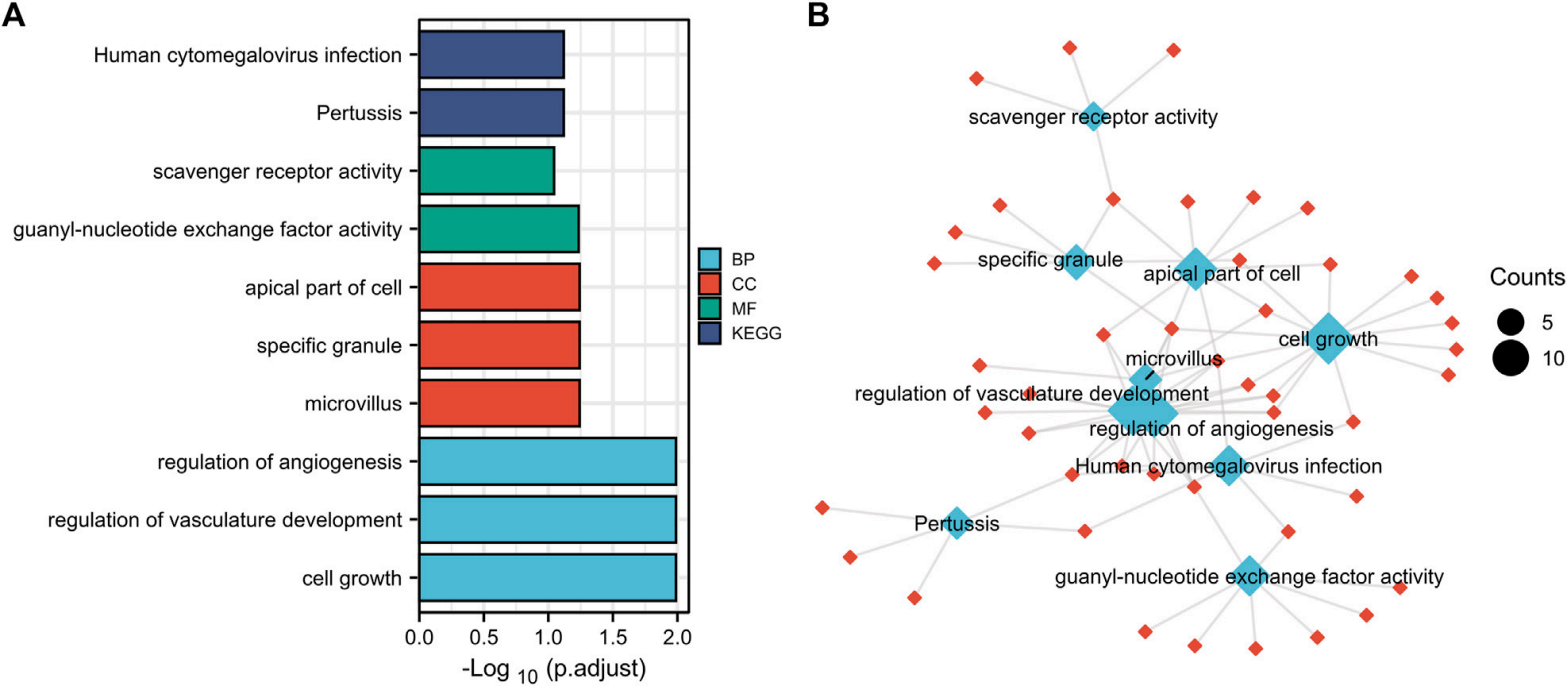
Function annotation of 144 common DEGs. **(A)**, GO terms (BP, MF, CC) and KEGG analysis of overlapped common DEGs (*p* < 0.01). GO, Gene Ontology. BP, biological process, MF, molecular function, CC, cellular component. KEGG, Kyoto Encyclopedia of Genes and Genomes. **(B)**, network of GO and KEGG.

Function annotation has been carried out among the 144 common DEGs (*p* < 0.01). BP category suggested that cell growth (*p* = 1.02E-05), regulation of vasculature development (*p* = 1.11E-05) and regulation of angiogenesis (*p* = 2.08E-05) were important process of the common DEGs (Figures 3A,B). MF results indicated these common DEGs were mostly involved in guanyl-nucleotide exchange factor activity (*p* = 0.00016) and scavenger receptor activity (*p* = 0.00049). The microvillus (*p* = 0.0003), specific granule (*p* = 0.001), apical part of cell (*p* = 0.0016) in CC category, indicating these genes play their roles in cell adhesion (Figures 3A,B). In addition, Pertussis (*p* = 0.00052) and Human cytomegalovirus infection (*p* = 0.00075) were significant pathways of common DEGs (Figures 3A,B). The GO and KEGG results were summarized in Supplementary Table S1.

### Protein-protein network and model analysis

**FIGURE 4:**
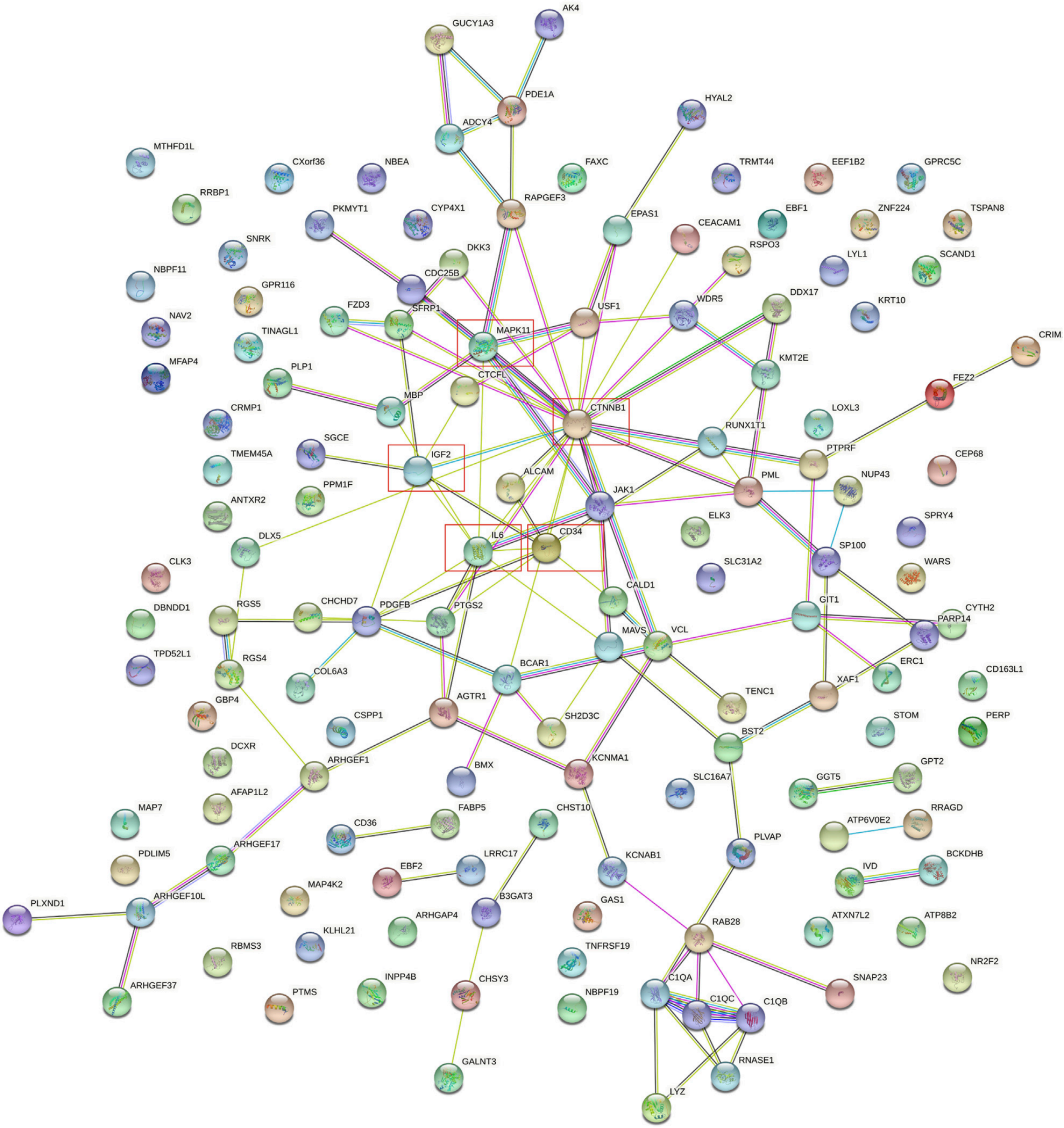
PPI network of 144 common DEGs.

**FIGURE 5:**
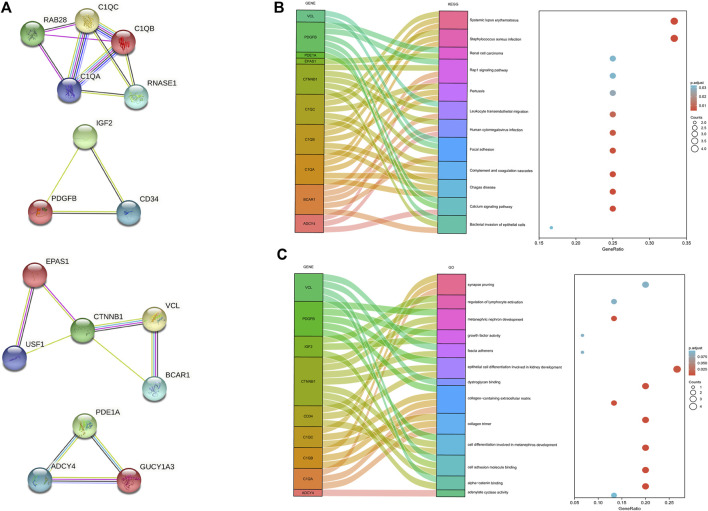
Significant gene module and function analysis of the model genes. **(A)**, Four significant gene clustering modules. **(B,C)**, GO and KEGG enrichment analysis of the model genes. The size of the circle represents the number of genes involved, and the abscissa represents the frequency of the genes involved in the term total genes.

The protein-protein interaction (PPI) network was constructed among 144 common DEGs to explore their potential interactions, including 668 nodes and 13484 edges (Figure 4). It is important to point out that many genes are individual. So, we selected four gene modules that interact most closely in PPI network were obtained through MCODE plugin of Cytoscape, which contained 2,534 edges involving 94 nodes. GO function analysis revealed that these model genes were mainly related to alpha-catenin binding, cell adhesion molecule binding, growth factor activity, synapse pruning, cell differentiation involved in metanephros development, fascia adherens and collagen trimer. KEGG results showed that these genes mainly involved in Focal adhesion, Pertussis and Bacterial invasion of epithelial cells (Figure 5).

### Selection of hub genes

**FIGURE 6:**
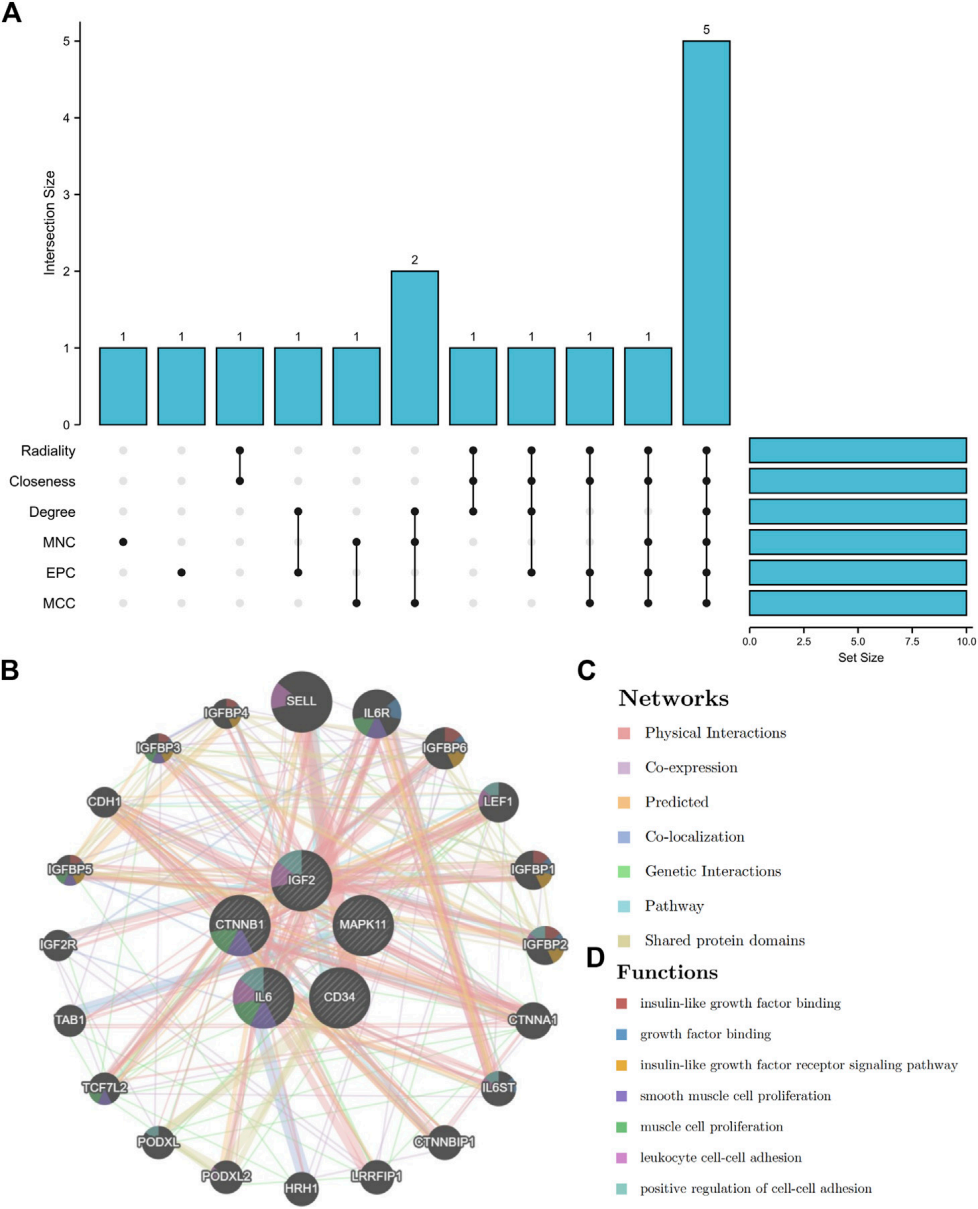
The UpSet diagram of hub genes and their co-expression network. **(A)**, six algorithms of cytoHubba selected 5 overlapped hub genes. **(B)**, Hub genes and their co-expression network.

By using six algorithms of cytoHubba, we identified the top 10 genes in our network (Figure 6A). After the selection, we found five overlapping hub genes which contained in six algorithms results, including CTNNB1, IL6, CD34, IGF2, MAPK11 (Table 1). Their full name and the function of encoding proteins are shown in Table 2. The co-expression network and functions of hub genes were analyzed using GeneMANIA database (Figure 6B). They showed a complex network with the, physical interactions of 77.6%, co-expression of 8.01%, co-localization of 3.63%, predicted of 5.37% and genetic interactions of 2.87%. Function analysis indicated that these genes mainly involved in insulin-like growth factor binding, leukocyte cell-cell adhesion and positive regulation of cell-cell adhesion (Figure 6C).

**TABLE 1:** Screening of hub genes using six algorithm in cytoHubba.

Rank	MCC	EPC	MNC	Degree	Closeness	Radiality
1	**CTNNB1**	**CTNNB1**	**CTNNB1**	**CTNNB1**	**CTNNB1**	**CTNNB1**
2	**IL6**	**IL6**	**IL6**	**IL6**	**IL6**	**IL6**
3	**CD34**	**CD34**	**MAPK11**	**CD34**	**CD34**	VCL
4	**IGF2**	**MAPK11**	**CD34**	**IGF2**	VCL	JAK1
5	C1QA	**IGF2**	C1QA	**MAPK11**	**MAPK11**	PTGS2
6	C1QB	JAK1	C1QB	C1QA	**IGF2**	**CD34**
6	**MAPK11**	PTGS2	**IGF2**	PML	JAK1	**MAPK11**
8	C1QC	PDGFB	JAK1	VCL	PTGS2	**IGF2**
9	JAK1	PML	C1QC	PDGFB	BCAR1	BCAR1
10	PTGS2	USF1	SFRP1	C1QB	PML	PML

**TABLE 2:** The gene symbol and function details of the hub genes.

Rank	Gene symbol	Total name	Function details
1	CTNNB1	Catenin Beta 1	The protein encoded by this gene is part of a complex of proteins that constitute adherens junctions (AJs). AJs are necessary for the creation and maintenance of epithelial cell layers by regulating cell growth and adhesion between cells. The encoded protein also anchors the actin cytoskeleton and may be responsible for transmitting the contact inhibition signal that causes cells to stop dividing once the epithelial sheet is complete. Finally, this protein binds to the product of the APC gene, which is mutated in adenomatous polyposis of the colon. Mutations in this gene are a cause of colorectal cancer (CRC), pilomatrixoma (PTR), medulloblastoma (MDB), and ovarian cancer. Alternative splicing results in multiple transcript variants
2	IL6	Interleukin 6	This gene encodes a cytokine that functions in inflammation and the maturation of B cells. In addition, the encoded protein has been shown to be an endogenous pyrogen capable of inducing fever in people with autoimmune diseases or infections. The protein is primarily produced at sites of acute and chronic inflammation, where it is secreted into the serum and induces a transcriptional inflammatory response through interleukin 6 receptor, alpha. The functioning of this gene is implicated in a wide variety of inflammation-associated disease states, including suspectibility to diabetes mellitus and systemic juvenile rheumatoid arthritis. Elevated levels of the encoded protein have been found in virus infections, including COVID-19 (disease caused by SARS-CoV-2)
3	CD34	CD34 Molecule	The protein encoded by this gene may play a role in the attachment of stem cells to the bone marrow extracellular matrix or to stromal cells. This single-pass membrane protein is highly glycosylated and phosphorylated by protein kinase C. Two transcript variants encoding different isoforms have been found for this gene
4	IGF2	Insulin Like Growth Factor 2	This gene encodes a member of the insulin family of polypeptide growth factors, which are involved in development and growth. It is an imprinted gene, expressed only from the paternal allele, and epigenetic changes at this locus are associated with Wilms tumour, Beckwith-Wiedemann syndrome, rhabdomyosarcoma, and Silver-Russell syndrome. A read-through INS-IGF2 gene exists, whose 5′ region overlaps the INS gene and the 3′ region overlaps this gene. Alternatively spliced transcript variants encoding different isoforms have been found for this gene
5	MAPK11	Mitogen-Activated Protein Kinase 11	This gene encodes a member of a family of protein kinases that are involved in the integration of biochemical signals for a wide variety of cellular processes, including cell proliferation, differentiation, transcriptional regulation, and development. The encoded protein can be activated by proinflammatory cytokines and environmental stresses through phosphorylation by mitogen activated protein kinase kinases (MKKs). Alternative splicing results in multiple transcript variants

### TF-mRNA-microRNA regulatory network analysis

**FIGURE 7:**
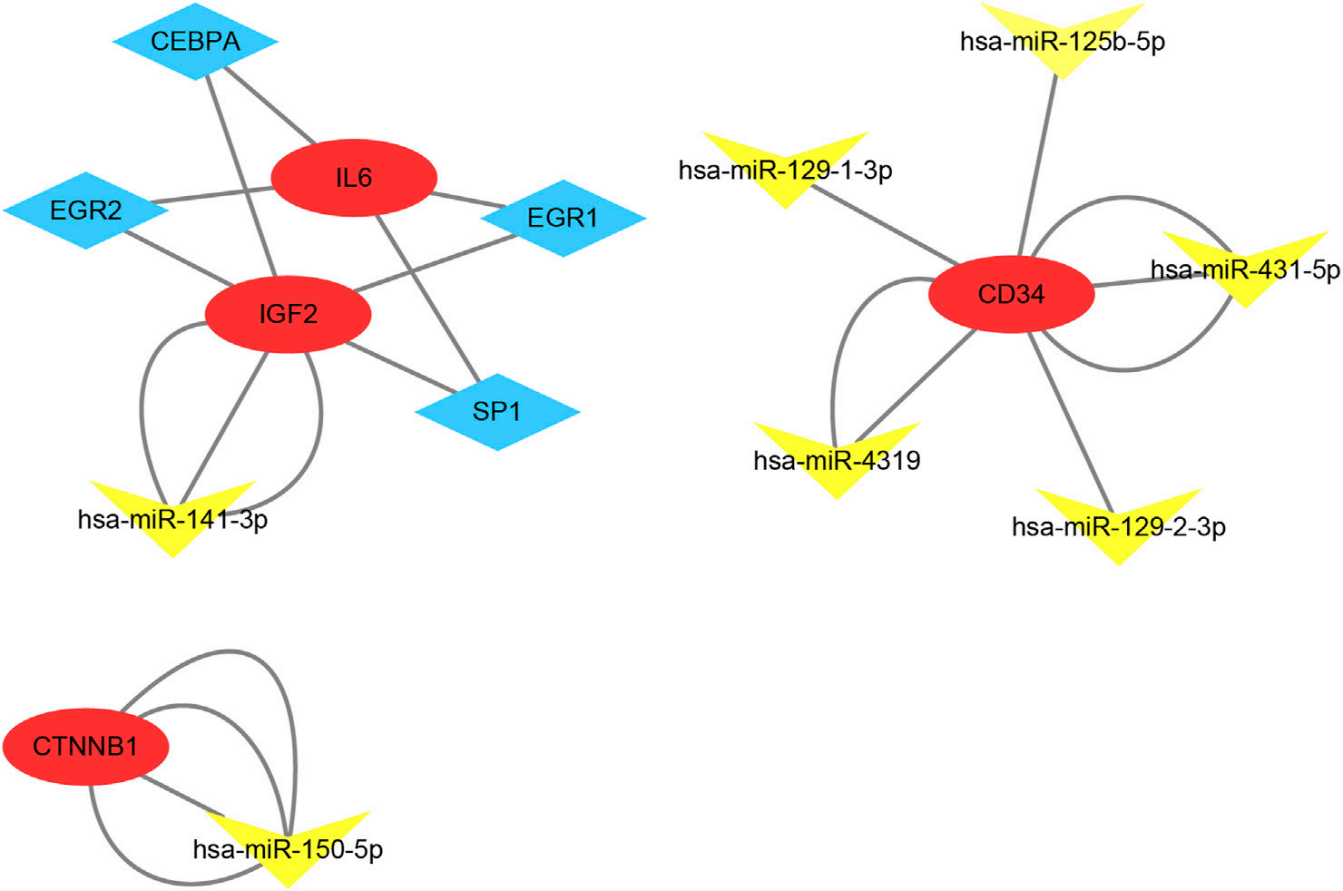
TF-mRNA-microRNA regulatory network. Red circles represent hub genes, blue diamonds represent transcription factors and yellow triangles represent microRNA.

According to the results in TRUUST and Mirwalk, 4 TFs and 7 microRNA have regulatory relationships with these hub genes (Figure 7). CEBPA, EGR2, EGR1 and SP1 were the predicted TFs of IL6 and IGF2 (Qvalue<0.01). In addition, hsa-miR-141-3p can target the 3′UTR of IGF2 (*p* = 0.01). hsa-miR-150-5p can target the 3′UTR of CTNNB1 (*p* = 0.01). Moreover, hsa-miR-431-5p, hsa-miR-4319, hsa-miR-129-1-3p, hsa-miR-125b-5p and hsa-miR-129-2-3p were the predicted microRNA of CD34 (*p* < 0.01).

### Validation of hub genes, TFs and microRNA expression

**FIGURE 8:**
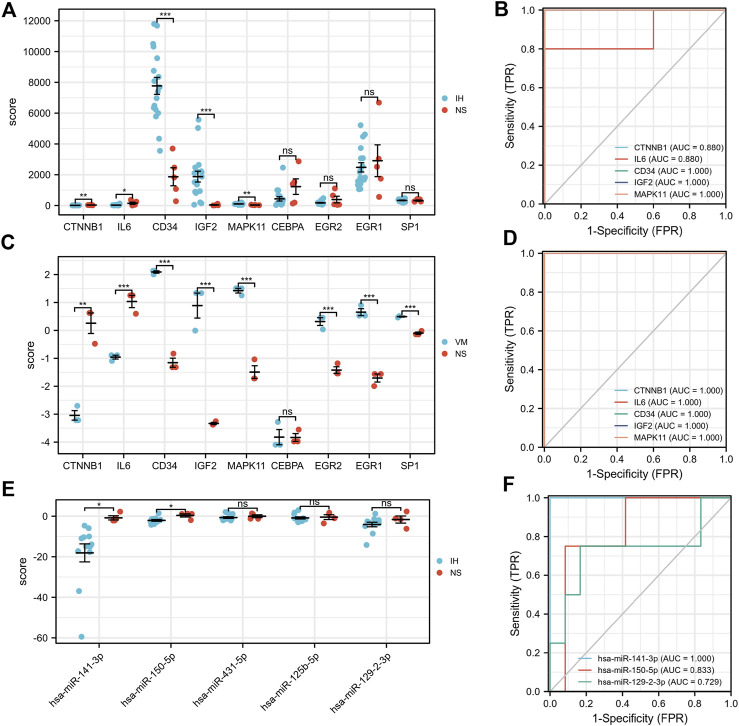
Validation of hub genes, TFs and microRNA expression and their ROC curves. *p < 0.05; **p < 0.01; ***p < 0.001.

Our results showed that CD34, IGF2, MAPK11 were significantly upregulated in IH patients (Figures 8A,C). In contrast, CTNNB1, IL6 were significantly downregulated. Moreover, the expressions of EGR2, EGR1, and SP1 were significantly upregulated in VM, but not significantly in IH. Additionally, has-miR-141-3p and has-miR-150-5p were significantly downregulated in IH (Figure 8E). All of the hub genes (AUC>0.8), has-miR-141-3p (AUC = 1) and has-miR-150-5p (AUC = 0.83) have shown good efficiency in distinguishing diseases from healthy people (Figures 8B,D,F).

## Discussion

Evidence suggests that IH and VM share some common pathogenic factors. However, their common differentially expressed mRNAs and the detailed molecular mechanisms remain unclear. In the present study, extensive bioinformatics methods were used to investigate the common DEGs of IH and VM, and to estimate the pathways involved in the hub genes. The *P*.Value <0.05 and LogFC (fold change) > 1 or < -1 were set as the cutoffs. At this threshold, there were total 989 common DEGs between IH and VM. In order to screen out the most significantly changed genes and narrow the range, we adjusted the threshold to adjP<0.05 and LogFC (fold change) > 1.5 or < -1.5. Then, we obtained 107 overlapping upregulated genes and 37 downregulated genes. Function enrichment analysis indicated that these common DEGs were mainly involved in cell growth, regulation of vasculature development and regulation of angiogenesis. The two most important cells in the course of IH are hemangioma stem cells and hemangioma endothelial cells. Infantile hemangioma is characterized by massive proliferation of hemangioma endothelial cells (Lv et al., 2022). The expression analysis indicates that VM endothelium is misspecified and hyperproliferative, suggesting that VMs are biologically active lesions (Lee et al., 2021). Abnormal proliferation and regulation of vascular endothelial cells can seriously affect vascular development and angiogenesis. Propranolol is a first-line drug for IH treatment and has an antiproliferative and cytotoxic against hemangioma endothelial and stem cells (Ma et al., 2021). These results strongly indicate that the abnormal in vasculature development and angiogenesis are jointly involved in the occurrence and development of these two diseases.

A total of five hub genes (CTNNB1, IL6, CD34, IGF2, MAPK11) were identified by constructing a PPI network. These genes occupy a central position in the PPI network. In addition, these hub genes also play a core role in module 2 and module 3, including CTNNB1, CD34, IGF2. The co-expression and function analysis indicated that these hub genes mainly involved in insulin-like growth factor binding and positive regulation of cell-cell adhesion. Among them, insulin-like growth factor receptor signaling pathway may be the core mechanism affecting vascular abnormalities. IGF2 is required for the continuous expansion of the feto-placental microvasculature in late pregnancy. The angiocrine effects of IGF2 on placental microvasculature expansion are mediated through IGF2R and angiopoietin-Tie2/TEK signaling. Additionally, IGF2 exerts IGF2R-ERK1/2-dependent pro-proliferative and angiogenic effects (Sandovici et al., 2022). CTNNB1 upregulates DLL4 transcription and strongly increases Notch signal in endothelial cells, leading to vascular abnormalities. Reduced Notch signaling in IH cells decreased cell proliferation, migration, and tumor formation (Ma et al., 2021). Many vascular malformations share similar aberrant molecular signaling pathways with cancers and inflammatory disorders (Pang et al., 2020). It is reported that inhibiting the IL-6/STAT3/HIF-1α signaling pathways could suppress IH growth (Maimaiti et al., 2022). These results indicated that the hub genes were involved in several signaling pathways mediated by growth factor and immune gene, which play a critical role in regulating endothelial cells. The regulation of vascular endothelial cell proliferation may be the common mechanism for the treatment of IH and VM. Furthermore, we made an in-depth analysis of the link between the hub gene and these two diseases.

CTNNB1 also named Catenin Beta 1. The protein encoded by CTNNB1 is part of a complex of proteins that constitute adherens junctions (AJs). AJs are necessary for the creation and maintenance of epithelial cell layers by regulating cell growth and adhesion between cells (Liu et al., 2022). Studies have shown that endothelial specific stabilization of Wnt/β-catenin signaling changes the early vascular development of embryos (Corada et al., 2010). β-catenin upregulates Dll4 transcription and strongly increases Notch signaling in the endothelium, resulting in loss of vascular remodeling, intersomatic vascular elongation, branch defect and loss of venous characteristics. Recently, Duan X et al. found that FOXC1A can regulate vascular integrity and brain vascular development through targeting CTNNB1 (Duan et al., 2022). In addition, CTNNB1 was also found to be closely related to a variety of tumor diseases such as liver cancer, gastrointestinal carcinoma and endometrial carcinoma (Ambrozkiewicz et al., 2022; Chao et al., 2022). Although few direct studies have shown the correlation between CTNNB1 and IH. We still insist that CTNNB1 is a potential biomarker strongly associated with the development and progression of IH and VM.

IL6 (interleukin 6) encodes a cytokine that functions in many autoimmune diseases or infections. It is reported that IL6 can regulate macrophage polarization controls atherosclerosis associated vascular intimal hyperplasia (Chen et al., 2022). Dritsoula et al. (2022) demonstrated that IL-6, through STAT3 phosphorylation, activates LRG1 transcription resulting in vascular destabilization. Here, studies have reported a predictive association between IL6 and subsequent cerebral cavernous malformation disease clinical activity (Girard et al., 2018).

IGF2 (insulin-like growth factor 2) is a member of the insulin family, which are involved in human development and growth. It has been detected to be highly expressed during the proliferating phase of IH, but the underlying mechanism is unclear (Yu et al., 2004). The same results can be found in Muller’s experiments that the serum levels of GLUT1, IGF-2, and VEGF-A in IH were significantly higher than those in healthy control (Aydin Köker et al., 2021). IGF2 can stimulate multiple steps of endothelial progenitor cells (EPC) homing *in vitro* and promote both EPC recruitment and incorporation into the neovascular area, resulting in enhanced angiogenesis (Maeng et al., 2009). Taken together, the utilization of the IGF2 system may facilitate the development of novel therapeutic approaches for IH and VM.

CD34 (hematopoietic progenitor cell antigen 34) plays an important role in the adhesion of stem cells to extracellular matrix or stromal cells. It is related to multiple diseases such as hemangiopericytoma, malignant and neurofibroma. Immunohistochemical staining showed that CD34 was positive in IH lesion cells (Johnson et al., 2018). *In vivo* experiments, miR-130a inhibition effectively suppressed the tumor growth by reducing the expression of angiogenic markers and the percentage of CD31^+^ and CD34^+^ to inhibit angiogenesis (Gao et al., 2017). The deletion of FOXF1 reduces the expression of endothelial genes that are essential for vascular development, such as CD34 (Ren et al., 2014). Therefore, regulating the expression of CD34 may be a potential method for effective treatment of IH and VM.

Mitogen-Activated Protein Kinase 11 (MAPK11) is involved in a variety of cellular processes, including cell proliferation, differentiation and development. Acute H (2) O (2) activation of MAPK11-p38 is the main cause of endothelial dysfunction during pregnancy (Chen et al., 2005). In addition, it has been reported that Ak1-MAPK11-cofilin signal is essential for the proliferation and neovascularization of mouse retinal endothelial cells induced by hypoxia (Kumar et al., 2016). No studies have reported the link between MAPK11 expression and IH and VM. Taken together, we consider that MAPK11 is a novel biomarker closely associated with vascular anomalies.

Finally, it is worth noting that all of the hub genes have shown good efficiency in distinguishing diseases from healthy people (AUC of ROC>0.8). Furthermore, we found that has-miR-141-3p and has-miR-150-5p significantly downregulated in IH patients. In our TF-mRNA-microRNA network, has-miR-141-3p can target 3′UTR of IGF2. Since their expression in IH is exactly opposite, we speculate that the has-miR-141-3p-IGF2 axis is a potential pathway regulating vascular abnormalities. In some cases, history and clinical presentation are not sufficient to diagnose vascular abnormalities, especially in infants and young children. Our results provide additional noninvasive biomarkers to help distinguish between the two diagnoses.

We must acknowledge the limitations in this study. First, the sample size of each group is slightly small. Second, further experimentally validation is required to explore the function and changes of hub genes and signaling pathways. These follow-up steps contribute to a deeper understanding of crosstalk between the two diseases.

## Conclusion

In conclusion, our study investigated the common DEGs and molecular mechanism in IH and VM. Base on PPI network analysis, five hub genes (CTNNB1, IL6, CD34, IGF2, MAPK11) were identified. Furthermore, we found that has-miR-141-3p and has-miR-150-5p significantly downregulated in IH patients. The regulation of vascular endothelial cell proliferation may be the common mechanism for the treatment of IH and VM. This study provides insight into the crosstalk of IH and VM and obtains several biomarkers relevant to the diagnosis and pathophysiology of vascular abnormalities.

## Data Availability

The original contributions presented in the study are included in the article/Supplementary Material, further inquiries can be directed to the corresponding authors.

## Abbreviations

GEO, Gene Expression Omnibus; DEGs, differentially expressed genes; PPI, protein−protein interaction; IH, infantile hemangiomas; NS, normal skin; VM, venous malformations; FDR, false discovery rate; FC, fold changes.
